# Spontaneous Infertility Secondary to Testicular Sarcoidosis: A Case Report

**DOI:** 10.7759/cureus.10165

**Published:** 2020-08-31

**Authors:** Holly A Bathen, Ellen Wood

**Affiliations:** 1 Research, Lake Erie College of Osteopathic Medicine, Bradenton, USA; 2 Reproductive Endocrinology and Infertility, IVFMD, Cooper City, USA

**Keywords:** infertility, sarcoidosis, azoospermia, testicular sarcoidosis, ivf, tesa, icsi

## Abstract

Sarcoidosis is a multisystem disease that can affect any region of the body. Rarely, sarcoid involvement may even involve the male genitourinary tract, including the testicles. Testicular sarcoidosis causes spontaneous and severe effects on male fertility due to obstructive azoospermia. The case presented offers an insight into successful fertility treatment in a patient with obstructive testicular sarcoidosis. The patient and his partner presented to the clinic two years post successful natural conception of their first child with subsequent infertility. Within this period, the male partner was diagnosed with sarcoidosis and was on a treatment plan consisting of methotrexate and glucocorticoids. Complete azoospermia was confirmed via two separate semen analyses six weeks apart. The patient’s testosterone (free and total), thyroid stimulating hormone (TSH), prolactin, follicle stimulating hormone (FSH), and luteinizing hormone (LH) were all within normal limits. With approval of pulmonology, methotrexate was discontinued for three months; however, subsequent semen analysis revealed no improvement. The patient was referred to urology, who confirmed the presence a palpable testicular nodule. Treatment of infertility was eventually achieved via testicular sperm aspiration (TESA) followed by in vitro fertilization (IVF) using intracytoplasmic sperm injection (ICSI). This treatment was successful in achieving one blastocyst and one morula, which were replaced via fresh transfer, resulting in a successful term singleton pregnancy. The possibility of obstructive azoospermia should be considered in males diagnosed with sarcoidosis who are seeking to preserve their reproductive potential.

## Introduction

Sarcoidosis is an inflammatory disease process that causes systemic non-caseating granuloma formations, typically presenting with thoracic lesions such as hilar lymphadenopathy [1]. There are no formal diagnostic criteria for sarcoidosis; however, evidence of pulmonary lesions, elevated levels of angiotensin converting enzyme (ACE), and biopsy of a non-caseating granuloma are the classic clinical picture. It also must be noted that it is necessary to rule out other granulomatous diseases such as tuberculosis in order to diagnose sarcoidosis [2]. However, in certain instances a more obscured presentation of sarcoidosis may develop due to the disease’s ability to affect multiple organ systems. The disease has been reported to have from neurological to cutaneous manifestations [2].

Obstructive azoospermia is the result of a blockage of the male reproductive tract, leading to a complete absence of sperm in the ejaculate and accounts for approximately 40% of all cases of azoospermia [3]. Obstruction may be congenital or acquired and may include one or more segments of the male reproductive tract: epididymis, vas deferens, and ejaculatory ducts. Acquired causes of obstructive azoospermia include vasectomy, infection, trauma, or iatrogenic injury. Testicular sarcoidosis is an incredibly rare cause of obstructive azoospermia with an incidence of 0.2% of testicular involvement in known sarcoidosis cases [1]. In this article, a case will be reviewed of a man who began with no fertility problems to completely azoospermic within two years of his diagnosis of sarcoidosis.

## Case presentation

A 35-year-old G3P0 woman presented to the reproductive endocrinology and infertility clinic hoping to conceive after three first-trimester spontaneous abortions. Each was easily conceived. The patient had a significant history of subseptate uterus and a 3-cm intramural fibroid. Her history was otherwise unremarkable. Her partner was healthy with no significant history and on no medications at the time. The uterine anomaly was felt to be the cause of her spontaneous abortions. The patient underwent laparoscopic myomectomy, removal of uterine septum, and ablation of stage 1. The fallopian tubes were patent bilaterally. Following recovery, the patient called to report a positive home pregnancy test. She carried the pregnancy to term and gave birth to a healthy infant via low transverse cesarean section.

Twenty seven months later, the patient presented with intention to conceive again. The patient had no changes in her history other than the cesarean. Her husband, however, had been diagnosed with sarcoidosis and was now taking solumedrol, methotrexate, and levaquin. A semen analysis was recommended at this point due to the recent diagnosis and medications.

Preconception labs were drawn, and the patient was advised to begin baby Aspirin and prenatal vitamins and attempt conception naturally. After nine months of unsuccessful attempts at natural conception, the couple returned for semen analysis. Semen analysis was conducted on two separate samples six weeks apart. Both samples revealed complete azoospermia. The couple was counseled on possible methotrexate-induced male factor infertility, and the male partner discontinued his treatment with methotrexate with pulmonary approval.

After discontinuing methotrexate for three months, a semen analysis was performed to reveal complete azoospermia. The patient’s testosterone (free and total), thyroid stimulating hormone (TSH), prolactin, follicle stimulating hormone (FSH), and luteinizing hormone (LH) were all within normal limits. The patient was referred to urology for consideration to use sperm from a testicular sperm aspiration (TESA) for in vitro fertilization (IVF).

TESA was determined to be an appropriate course of treatment. Examination by the urologist revealed a palpable testicular nodule on the day of sperm aspiration, which was identified as a sarcoid nodule by the urologist. The couple underwent a fresh IVF cycle using the fresh testicular sperm.

Twenty-five swimming sperms were initially found in the supernatant and many more twitching. Six vials of testicular tissue were also cryopreserved. Sixteen oocytes were retrieved at transvaginal oocyte retrieval (TVOR), and seven mature oocytes underwent intracytoplasmic sperm injection (ICSI). Two oocytes fertilized normally. Embryo transfer occurred five days after TVOR, and one blastocyst and one compacting morula were replaced. The patient successfully conceived a singleton pregnancy and delivered a healthy male infant at term.

## Discussion

Azoospermia is present in approximately 10% of infertile men and about 1% of men in the general population [3]. The workup for azoospermia should be divided into three categories: pre-testicular, testicular, or post-testicular dysfunction. Pre-testicular refers to hypothalamic-pituitary dysfunction, whereas post-testicular most commonly refers to obstruction or ejaculatory dysfunction [4]. The most common causes of azoospermia are testicular failure and obstruction [3]. In this specific case, the patient did not have a baseline semen analysis performed; however, it is evident from the four pregnancies from his semen that he was not azoospermic prior to his diagnosis with sarcoidosis. Due to this, it was reasonable to rule out genetic factors such as gene mutations, Y chromosome microdeletions, or sex chromosome abnormalities as the primary cause of the new onset infertility. The patient had also been on methotrexate for two years prior to the original two semen analyses that diagnosed the azoospermia. The current consensus regarding methotrexate use and male fertility is unclear, and the recommendation to stop methotrexate use for three months, while not unsafe, is based on the concept of new spermatogenesis rather than evidence of teratogenicity associated with the drug [5]. In this case, the patient remained azoospermic even following trial of discontinuation of methotrexate, prompting further evaluation. Hormone levels including testosterone, LH, and FSH were drawn, and all were within normal limits, and therefore the differential narrows to an obstructive pattern. A limitation to this case is the clinical diagnosis of an obstructive sarcoid nodule. Due to loss of follow-up, a confirmatory biopsy of the testicular nodule was never reported by the urology team following the TESA.

There are very few cases on genitourinary involvement of sarcoidosis currently in the literature. In one case, there was reported improvement of azoospermia due to testicular and epididymal involvement after treatment with high-dose prednisolone therapy [6]. The study retrospectively analyzed sperm concentration before and after five months of treatment [6]. The steroids increased the sperm count from 0 to 1.2 million/mL [6]. In our case, however, the patient was already on solumedrol for two years when he presented with azoospermia.

A similar case to the one presented in this report was published by Svetec et al. in 1998 [7]. A 36-year-old male who was previously fertile, fathering three children with the latest being only one year prior, presented with infertility following the diagnosis of sarcoidosis [7]. His case was followed for ten years, which showed an unpredictable relapsing and remitting oligo to azoospermia that was unresponsive to steroids [7]. It was actually suggested in this study that all men diagnosed with sarcoidosis should consider baseline semen analysis and sperm banking upon diagnosis due to the unpredictable nature of the disease [7]. At the time of publishing, this patient was undergoing evaluation for epididymal aspiration and ICSI [7].

In 2012, a novel case report was published by Kovac et al. [8]. This case outlined the first case of sarcoidosis diagnosed upon presentation of infertility secondary to azoospermia [8]. This patient, similar to the one reported in this paper, fathered two children prior to the infertility [8]. His LH, FSH, testosterone, estradiol, and prolactin were all within normal range [8]. Due to his normal hormone levels in conjunction with normal volume azoospermia, a testicular biopsy was conducted and demonstrated granulomas consistent with sarcoidosis [8]. This patient was able to conceive naturally following steroid treatments [8]. Since this case describing infertility as the presenting complaint of sarcoidosis [8], a subsequent case has been published by Albayrak et al. [9]. In this case, the patient was found to have pulmonary involvement after diagnosis of testicular lesions due to complaint of a chronic cough [9]. The authors argue that genitourinary sarcoidosis should always be considered in male infertility patients with systemic complaints [9]. Furthermore, a report published in 2008 incidentally diagnosed reduced fertility secondary to testicular involvement of sarcoid by 67-gallium-citrate scintigram with no symptoms or palpable mass [10]. These cases highlight the subtlety of testicular sarcoid involvement and suggest a need for further studies on prevalence of genitourinary nodules in asymptomatic sarcoidosis patients.

## Conclusions

This case report offers an insight into successful fertility treatment of methotrexate and steroid-resistant testicular sarcoidosis. Although the genitourinary system involvement is seldom reported in sarcoidosis patients, it has been shown through these reports to have spontaneous and severe effects on male fertility. These cases illustrate that sarcoid involvement of the genitourinary organs in males may be extremely subtle, relapsing and remitting, or even sub-clinical when patients are not attempting pregnancy. Despite the low incidence of genitourinary sarcoid, screening male patients with an initial semen analysis upon diagnosis of sarcoidosis would be a low-cost and effective measure to both screen and evaluate for future genitourinary involvement. Additionally, we suggest subsequent studies to confirm the low prevalence of genitourinary involvement due to the elusive nature of the disease. This case serves to add to the literature on the rare diagnosis of testicular sarcoidosis and the successful infertility management of that patient.
